# Gender-based difference in early mortality among patients with ST-segment elevation myocardial infarction: insights from Kermanshah STEMI Registry

**DOI:** 10.34172/jcvtr.2020.10

**Published:** 2020-02-19

**Authors:** Soraya Siabani, Patricia M Davidson, Maryam Babakhani, Nahid Salehi, Yousef Rahmani, Farid Najafi, Hossein Karim, Ali Soroush, Behrooz Hamzeh, Mojtaba Amiri, Hossein Siabani

**Affiliations:** ^1^Cardiovascular Research Center, Health Institute, Kermanshah University of Medical Sciences, Kermanshah, Iran; ^2^University of Technology Sydney, Sydney, NSW, Australia; ^3^The Johns Hopkins University, Baltimore, MD, USA

**Keywords:** Myocardial Infarction, Sex, Mortality, Registry, Cohort

## Abstract

***Introduction:*** This study aimed to evaluate the in-hospital mortality of patients with ST-segment elevation myocardial infarction (STEMI), according to gender and other likely risk factors.

*** Methods:*** This study reports on data relating to 1,484 consecutive patients with STEMI registered from June 2016 to May 2018 in the Western Iran STEMI Registry. Data were collected using a standardized case report developed by the European Observational Registry Program (EORP). The relationship between in-hospital mortality and potential predicting variables was assessed multivariable logistic regression. Differences between groups in mortality rates were compared using chi-square tests and independent t-tests.

*** Results:*** Out of the 1484 patients, 311(21%) were female. Women were different from men in terms of age (65.8 vs. 59), prevalence of hypertension (HTN) (63.7% vs. 35.4%), diabetes mellitus (DM) (37.7% vs. 16.2%), hypercholesterolemia (36.7% vs. 18.5%) and the history of previous congestive heart failure (CHF) (6.6% vs. 3.0%). Smoking was more prevalent among men (55.9% vs. 13.2%). Although the in-hospital mortality rate was higher in women (11.6% vs. 5.5%), after adjusting for other risk factors, female sex was not an independent predictor for in-hospital mortality. Multivariable analysis identified that age and higher Killip class (≥II) were significantly associated with in-hospital mortality rate.

***Conclusion:*** In-hospital mortality after STEMI in women was higher than men. However, the role of sex as an independent predictor of mortality disappeared in regression analysis. The gender based difference in in-hospital mortality after STEMI may be related to the poorer cardiovascular disease (CVD) risk factor profile of the women.

## Introduction

Acute coronary syndrome (ACS), including myocardial infarction (MI), is a costly condition and the leading cause of mortality in both women and men throughout the world.^1,2^ The incidence of MI is constantly increasing worldwide, mostly due to population aging and sedentary lifestyle.^3,4^ Many risk factors, directly or indirectly, may affect the outcomes of patients with MI.^5^ The outcomes disparity between women and men after acute myocardial infarction (AMI) has been reported frequently but with a wide variety in different ethnic and demographic groups.^5-9^ The reasons for the sex-based differences have not been clear yet, though, the known risk factors for MI such as diabetes mellitus (DM), hypercholesterolemia, and hypertension (HTN) in women are more frequent than men.^10-12^ After adjustment for possible confounders such as age and other risk factors, some researchers found no differences in the mortality rate between men and women,^9,12,13^ while some others showed that mortality remained higher in women.^14,15^ Hence, female gender might be a potential risk factor for mortality after acute MI which needs more investigation.^16^

The aim of this study was to evaluate the baseline and clinical characteristics of patients who died after ST-segment elevation myocardial infarction (STEMI). In addition, we investigated whether potential disparity in mortality between women and men with STEMI could be explained by gender as an independent risk factor.

## Materials and Methods

### Study population

This study is a part of an ongoing registry of consecutive patients with STEMI in Imam Ali Cardiovascular Center, Kermanshah University of Medical Sciences (KUMS). This is the main cardiovascular center in western Iran covering about two millions population mostly Kurdish with Caucasian race. Data adjudicated by the standards of the European Observational Research Program (EORP). Also we used the Standard for Reporting Qualitative Research (SRQR) guidelines.

Between July 1, 2017, and May 1, 2018, we evaluated patients admitted to the center with a presumed diagnosis of STEMI. One criterion for inclusion in the study was age ≥18 years old. Another inclusion criterion was the diagnosis of STEMI accompanied by the following criteria was required: chest pain or equivalent symptoms of more than 20 minutes duration within the last 24 hours before admission and, electrocardiographic changes consistent with new or presumed new ST-segment elevations or left bundle branch block, according to third universal definition of myocardial infarction defined by the European Society of Cardiology/ACCF/AHA/World Heart Federation Task Force for the Universal Definition of Myocardial Infarction.^17^ STEMI must be primary diagnosed by the emergency medical doctors. Data collection and validation of the diagnosis was then supervised by a physician who was responsible for the quality control of the project.

Patients were excluded from the study if they developed STEMI after admission in the hospital, or developed STEMI after percutaneous coronary angioplasty or coronary bypass surgery and/or hospitalized in another hospital more than 24 hours before coming to Imam Ali hospital, or who did not sign the consent form to participate in the study.

### Data collection

Using case report forms, data were collected by a nurse and a research assistant working in the Kermanshah Cardiovascular Research Center (KCRC), who were trained in uniform protocols for patient entry and data gathering. Case report form was developed by the EORP. All completed case report forms were verified and checked for errors by the quality control physician before submission for final analysis, and then forwarded online to the EORP website. We used patients’ national identification numbers to avoid duplicate admissions to the registry. The collected data included demographic characteristics (e.g. age), clinical histories (e.g. previous myocardial infarction), admission process (e.g. presenting symptoms), biochemical and electrocardiographic findings (e.g. blood sugar), treatment procedures (e.g. percutaneous coronary intervention, PCI), in-hospital outcomes (e.g. vital status). Standardized definitions of all variables (e.g. clinical diagnoses) were used. Likewise, mortality data (in-hospital) were obtained for all included cases.

### Statistical methods

Baseline data including demographic characteristics were compared between men and women. Data were described using mean ± standard deviation (SD) if they were normally distributed; otherwise, frequencies and percentages were reported. Differences between subgroups were assessed using independent *t* tests for continuous and normally distributed variables and chi-square (or Fisher exact tests) for other variables. We compared the in-hospital mortality rates among women and men and assessed the independent role of sex in patients’ mortality using multivariable logistic regression models. Odds ratios (ORs) and 95% confidence Intervals (CIs) were calculated. The body mass index (BMI) ≥25, HTN, DM, current smoking, hypercholesterolemia, congestive heart failure (CHF), Killip class (at first presentation) ≥ II, symptom-to-balloon (STB) time> 360 minutes and door-to-balloon (DTB) time > 90 minutes (18) used as covariates, while the dependent variable was in-hospital mortality. A test was considered statistically significant if the probability value (*P* value) was less than 0.05. The Statistical Package for Social Science (SPSS) software, version 23 (IBM Corp., Armonk, NY USA) was used for analyzing data.

### Definition

STB time was defined as the time between the onset of symptoms to first balloon inflation or first device delivery in the culprit vessel.

DTB time was defined as the time between patient’s arrival at the PCI hospital and the first balloon inflation or first device delivery in the culprit vessel.

## Results

During 20 months, a total of 1484 patients, 1173 (79%) men, and 311 (21%) women met the inclusion criteria for this study. A significant difference in presenting clinical characteristics based on sex was observed. Women were, on average, older than men patients: the mean age of the women was 65.79 ± 11.34 years, whereas for the men was 59.04 ± 12.39 years (*P* = 0.02). The prevalence of hypercholesterolemia, DM, HTN and prior CHF was significantly higher in women compared to men, whereas more men were current smoker (Table 1). There was no statistically significant difference in the Killip classifications ≥П between men and women.

**Table 1:** The characteristics of patients with STEMI stratiﬁed by sex

**Variable**	**Men (n=1173)**		**Women (n=311)**		***P*** **value**
Age (y)^*^	59.04±12.39	65.79±11.34	0.02
BMI (kg/m^2^)^*^	25.97±3.73	26.84±4.30	0.001
Current smoker	655 (55.9)	41 (13.2)	0.001
DM	187 (16.2)	114 (37.7)	0.001
Hypercholesterolemia	208 (18.5)	110 (36.7)	0.001
HTN	410 (35.4)	195 (63.7)	0.001
Prior CHF	33 (3.0)	18 (6.6)	0.001
Killip class ≥П	99 (8.0)	25 (7.8)	0.89
Primary PCI performed	697 (59.40)	181 (58.20)	0.50
DTB Time (min)	111.58±120.30	126.86±144.93	0.49
STB Time (min)	250.30±423.70	289.83±290.40	0.86

BMI, body mass index; CHF, congestive heart failure; PCI, percutaneous coronary intervention; DTB, Door-to-Balloon; STB, Symptom-to-balloon; DM, diabetes mellitus. * Continuous variables expressed as mean ± SD, otherwise No. (%).

Primary PCI was performed for 878 patients (59.2%), 697 men (59.4%) and 181 (58.2%) women. The median DTB time and STB time (or treatment delay) were slightly longer in women than men, but the differences were not statistically significant (*P* ≥ 0.05) (Table 1).

The mean age of patients, at death, was 66.11±11.42 for men vs 67.36 ± 9.69 for women. In-hospital mortality rates (death before discharge from hospital) were 5.5% (64 out of 1173) for men and 11.6% (36 out of 311) for women (*P* = 0.001). Other sex-based differences in personal and clinical characteristics of patients were shown in Table 1.

The role of sex in in-hospital mortality from STEMI was further examined using logistic regression model. The sex-based difference in the mortality rates was disappeared in regression analysis after adjustment for the explanatory variables listed in Table 1 (OR 0.896, 95% CI 1.51- 1.73, *P* = 0.77).

Factors contributing to in-hospital mortality were analyzed separately for women and men. For both sexes, the most important factor that increased in-hospital mortality was Killip class≥ II (OR 21.94, 95% CI 1.28- 373.73, *P* = 0.03) for women vs. (OR 9.71, 95% CI 2.55- 36.92, *P* = 0.001) for men. Age was significantly associated with the in-hospital mortality in men (OR: 1.05, 95% CI 1.00-1.12, *P* = 0.04, but not in women (OR: 1.00 (0.91-1.10, *P* =0.92) (Table 2).

**Table 2:** Multivariate analysis of in-hospital mortality according to sex

**Risk factor**	**Male( n=64)**		**Female( n=36)**	
	**OR (95 % CI**	***P*** **value**	**OR (95 % CI**	***P*** **value**
Age	1.05 (1.00-1.12)	0.04	1.00 (0.91-1.10)	0.92
BMI	1.51 (0.41-5.50)	0.53	1.37(0.18-10.54)	0.75
Current smoker	0.93 (0.25-3.37)	0.91	0.32 (0.01-6.54)	0.46
DM	1.33 (0.25-7.04)	0.73	0.47 (0.04-5.15)	0.54
Hypercholesterolemia	0.31 (0.03-2.80)	0.30	3.29 (0.41-26.04)	0.25
HTN	1.61 (0.45-5.71)	0.45	0.46 (0.06-3.30)	0.44
Previous CHF	0.00 (0.00-0.01)	0.99	2.71 (0.17-42.91)	0.47
Killip class≥ П	9.71 (2.55-36.92)	0.001	21.94 (1.28-37.73)	0.03
DTB time	0.80 (0.23-2.07)	0.73	2.90 (0.27-30.33)	0.37
STB time	3.00 (0.72-12.40)	0.12	0.78 (0.06-9.18)	0.85

N, number; OR, odd ratio; CI, confidence interval; BMI, body mass index; CHF, congestive heart failure; DTB, door-to-balloon; STB, symptom-to-balloon; DM, diabetes mellitus.

## Discussion

The results of this study illustrated that, in western Iran, women who presented with STEMI were significantly older and had higher prevalence of cardiovascular risk factors i.e. diabetes, hypercholesterolemia, HTN, previous CHF, but were less likely to be current smokers compared to their men counterparts. Although STEMI was managed similarly in both sexes, the in-hospital mortality rate for women was two times more than men. However, after adjustment for other characteristics such as DM and HTN, early mortality was similar for women and men.

The fact that women present at an older age in the current study is consistent with the findings from other studies which have reported a mean age range of 58.8 to 60.9 years for men and 65.5 to 67.5 years for women.^6,10,18,19^ Furthermore, the higher incidences of DM, HTN, hypercholesterolemia, and prior CHF in women have been described in previous observations.^20,21^ Also, an international study reported that women were significantly older than men at the time of STEMI and had higher rates of HTN, DM, previous CHF, and hypercholesterolemia.^22^ Likewise, Birkemeyer et al reported that women were significantly older, with a higher prevalence of HTN and DM, and men were more likely to be current smokers.^23^

Also, consistent with some other reports,^24,25^ this study found no significant differences in treatment with primary PCI for women and men; however, DTB and STB were delayed slightly in women compared to men. These results support the findings of previous studies that women typically experienced a delay in DTB and STB.^26^ In fact, the potential reasons why women delay in seeking treatment may be due to their unawareness of cardiac disease symptoms or imputing their symptoms to cardiac causes.^27^ Patients education campaigns and programs, especially those targeted the women, can be cost-benefit in improving women’s awareness. Other reasons could be that women, particularly in developing countries, are often dependent to men financially, and in some societies the decision to seek treatment for women is made by men.^28^

In this study, although women’s mortality was higher than men, sex was not an independent predictor of in-hospital mortality at multivariate analysis. This result also concurs with the findings of previous studies. Barbosa et al reported that despite a high in-hospital mortality rate among women, sex was not an independent predictor of death in STEMI.^6^ Norris and colleagues found that compared to men, women with STEMI showed a higher rate of in-hospital mortality. They attributed this difference to women’s older age, sex clinical severity, and more comorbidities.^29^ Investigating 1548 cases of STEMI, De Luca and colleagues reported that female sex was not an independent prognostic factor of in-hospital mortality.^30^ In our study, the higher mortality reported among women may be due to their significantly higher rates of comorbidities, higher baseline risks, older age, and longer DTB and SBT, not associated to sex alone, which is consistent with the results of earlier studies.^31,32^ Indeed, poorer risk profile increases in-hospital mortality rates, but the risk of mortality remain the same for both genders when other factors are adjusted for.^33^

Nevertheless, some researchers have reported different results. For instance, Benamer et al described the results of a large French STEMI registry in which short term (in-hospital) mortality was higher in women and the impact of female sex on the mortality rate was significant.^20^ Also, Hersi and colleagues’ survey in Saudi Arabia showed that female gender was an independent risk factor for in-hospital mortality in STEMI.^24^ Likewise, in a study conducted by Lawesson et al, female sex was independently linked to higher in-hospital mortality.^34^ Differences in inclusion criteria and patients’ selection, sample size, clinical and socio-demographic characteristics, and treatment strategies might unfold these different results.

In addition, we found that Killip class ≥II and age were significant independent predictors of in-hospital mortality in both genders the results that are consistent with findings of pervious research. Reporting the short term mortality in a population of 1548 STEMI patients treated with primary PCI, De Luca et al found that Killip class ≥ II was associated with higher in-hospital mortality rates in both genders.^30^ This conclusion was also confirmed by DeGeare and colleagues who studied 2654 cases of AMI and found that higher Killip class was associated with greater in-hospital mortality rates in both genders.^35^ Similarly, El-Menyar et al reported that Killip class≥ II was an independent predictor of in-hospital mortality rate in the STEMI patients.^36^

Furthermore, our results demonstrated that old age was independently related to higher in-hospital mortality in men. This result is consistent with the finding of Waldecker et al, who showed that male patients had an age-related increase in in-hospital mortality rate.^37^

In fact, the impact of sex on mortality rate in STEMI is still controversial. Disease registries, especially hospital-based registries, are valuable sources providing more reliable information about the epidemiology of cardiovascular disease (CVD).^38^ Thus, this STEMI registry may help to prioritize research projects and interventions and help policy makers to develop health programs. It may result in improving STEMI patients’ health, assists health caregivers to revise their medical services, and estimate the burden of STEMI. The gender-based differences in in-hospital mortality in STEMI, may indicate gender based differences in access and use of health care services, which warrant further considerations.

### Limitations

The most important limitation of the current study, as an observational registry, was that such studies have non-randomized nature and may not be able to control for the effects of cofactors, although the researchers measured and controlled for the effects of main confounding factors. Furthermore, our data were derived from a single center registry; hence, our participants may not be the representative of the whole STEMI population.

## Conclusion

In-hospital mortality was higher in women with STEMI than their men counterparts. However, sex alone, was not a significant predictor of in-hospital mortality in these patients. The gender based differences in in-hospital mortality may be explained by the more adverse clinical risk profile in women. In general, access and use of health care programs need to be improved for women, and their CVD risk factors, in particular diabetes, HTN, and hypercholesterolemia be modified to in timely manner to reduce the gender based gaps in cardiovascular health outcomes.

## Competing interests

The authors declare that there were no conflicts of interest. In addition, the authors have no financial gain related to any aspect of the study.

## Ethical approval

The Research Ethics Committee at KUMS approved the study protocol (Ethics No. KUMS.REC.1395.252). Also, patients were informed about participating in the registry and signed the consent form. Patient data were kept confidential with the access limited to two of researchers and the quality control physician.

## Funding

This study was supported by Kermanshah University of Medical Sciences.

## Acknowledgments

We would like to thank the Kermanshah University of Medical Sciences for funding this project and express our sincere appreciation for the high quality collaboration of the Kermanshah Cardiovascular Research Center. We would like to thank the participants in the study.
